# Assessment of PRRSV and PCV2 seroprevalence and antigen prevalence in minipigs at laboratory-animal production facilities

**DOI:** 10.5455/javar.2024.k852

**Published:** 2024-12-28

**Authors:** Yoon Beom Lee, Ji Woon Kim, Woori Jo, Tae-Ku Kang, MinKyoung Sung, KilSoo Kim, Na-Hye Park, Gwang-Hoon Lee

**Affiliations:** 1 Preclinical Research Center, Daegu-Gyeongbuk Medical Innovation Foundation, Daegu, Republic of Korea; 2 Department of Veterinary Toxicology, College of Veterinary Medicine, Jyungpook National University, Daegu, Republic of Korea

**Keywords:** Experimental animal, Production facilities, Minipigs, Porcine circovirus type 2, Porcine reproductive and respiratory syndrome virus

## Abstract

**Objective:**

Pigs are used in various biomedical research fields because of their anatomical and physiological similarities to humans. While farm pigs are raised outdoors for several months, minipigs are typically raised in indoor barrier facilities for several years. Although numerous studies have investigated the prevalence of major pathogens, including porcine reproductive and respiratory syndrome virus (PRRSV) and porcine circovirus type 2 (PCV2), in farm pigs, similar studies targeting minipigs are lacking.

**Materials and Methods:**

We imported 57 minipigs to our institution from three experimental animal production facilities and immediately assessed the serological and antigenic prevalence of PRRSV and PCV2.

**Results:**

PRRSV and PCV2 had seroprevalences of 80.7% and 94.7%, respectively, with 0% antigen positivity rates for PRRSV types 1 and 2 and high-pathogenic PRRSV and PCV2.

Two factors could account for the high seroprevalence rates: the majority of individuals may have been vaccinated despite official claims from the origin facilities or exposed to PRRSV and PCV2. Preventing microbial infections is crucial for obtaining accurate and reproducible results.

**Conclusion:**

As the first investigation of microbial prevalence in minipigs, our findings indicate that minipigs raised in barrier facilities are not necessarily free from vaccines or infections. These results will significantly enhance the credibility of future biomedical research.

## Introduction

Animal research facilities are dedicated to scientific exploration and experimentation involving animals and serve various disciplines, such as medicine, biology, and life sciences. A wide array of animal species has been employed for research purposes, including mice, rats, rabbits, dogs, pigs, and monkeys [1,2]. Pigs are particularly noteworthy because of their anatomical and physiological resemblance to humans. They are used to assess the efficacy of drugs for cardiovascular diseases, ocular diseases, skin disorders, reproductive diseases, and even organ transplantation studies because their organs are similar in size to human organs. Additionally, because pigs have dimensions similar to those of humans, they have been used in diverse preclinical evaluations of medical devices [3,4]. However, laboratory pigs are very different from farm pigs in terms of their size and rearing environment.

Farm pigs are raised for consumption, can weigh more than 300 kg, and are typically reared for 6 months before being slaughtered at weights ranging from 100 to 120 kg [5,6]. In contrast, experimental pigs (referred to as minipigs) can be nurtured for prolonged durations and generally achieve weights between 30 and 70 kg by 2 years of age, making them substantially different from farm pigs [6,7]. Furthermore, farm pigs are reared in outdoor farm environments, whereas minipigs are housed indoors in controlled facilities. These facilities employ HEPA filters for air purification, and the environmental conditions are maintained at approximately 23°C ± 3°C and 50 ± 10% humidity. Minipigs are provided with sterilized feed and filtered water to ensure their health and protect them from microbial contaminants. Such stringent cleanliness measures safeguard the health of minipigs, allowing for controlled research variables and ensuring the accuracy and reliability of the experimental outcomes, crucial for evaluating the efficacy of pharmaceuticals and medical devices [8].

Porcine reproductive and respiratory syndrome virus (PRRSV) and porcine circovirus (PCV) are significant diseases affecting pigs worldwide, including Korea, where the swine industry is prominent. Because these diseases are particularly important in areas where the pig industry is developed and difficult to control, each authority of the countries is making efforts to manage and prevent them [9,10]. PRRSV was initially identified in North America (type 2) in the late 1980s and Europe (type 1) in the early 1990s, with nucleotide sequence similarities between the two types ranging from 55% to 70%, highlighting their substantial differences. In Korea, PRRSV types 1 and 2 cocirculate [11].

PRRSV is a positive-stranded RNA virus categorized under the family Arteriviridae within the order *Nidovirales*. It is characterized by reproductive disorders and respiratory issues and occurs in two main genotypes: the European prototype (EU-type, type 1), represented by the Lelystad virus, and the North American prototype (NA-type, type 2), identified as VR-2332 [12]. The Circoviridae family comprises small, icosahedral, nonenveloped viruses with single-stranded circular DNA genomes. Within the Circoviridae family, two genera (Gyroviruses and Circoviruses) are distinguished based on differences in genome size and organization. PCV types 1 and 2 (PCV1 and porcine circovirus type 2 (PCV2), respectively) belong to the genus Circovirus. PCV1 is considered nonpathogenic and is not linked to naturally occurring diseases, whereas PCV2 is the primary pathogen responsible for post-weaning multisystemic wasting syndrome [13,14].

According to previous studies, the antigen prevalence of PRRSV was approximately 69.8% in farm pigs in Korea from 2018 to 2022 [15], whereas in 2022, the antigen prevalence of PCV2 was 62.3% in farm pigs [16], indicating that PRRSV and PCV2 are prevalent diseases in farm pigs. Although numerous studies have explored the prevalence rates of PRRSV and PCV2 in farm pigs, no studies have explored microbial infections, such as PRRSV and PCV2, in minipigs maintained in experimental animal production facilities with barrier systems [11,16-20].

The health status of experimental animals significantly affects the reproducibility and accuracy of research outcomes. Microbial infections can alter the physiological state of animals and activate the immune system, potentially biasing experimental results. Moreover, infected animals can transmit infections to others during the research period, emphasizing the importance of maintaining animal health and minimizing microbial infections to enhance research reliability. Therefore, appropriate surveillance and preventive measures are required.

Our study aimed to evaluate the seroprevalence of PRRSV and PCV2 in pig populations from experimental animal production facilities to evaluate the immune status of these pigs. Additionally, PCR testing was used to investigate the antigen positivity rates in individuals with a positive antibody status and to ascertain whether they had been vaccinated or infected. Specifically, we sought to obtain information on the antibody and antigen positivity rates of pigs in controlled environments in experimental animal production facilities to enhance the accuracy and reliability of the experimental results.

## Materials and Methods

### Ethical approval

The animal study protocol was approved by the Institutional Review Board of the Daegu Gyeongbuk Medical Innovation Foundation (protocol code: KMEDI-24032701; approval date: 03 March 2024).

### Isolation of serum

From 2021 to 2024, pigs (*n = *57) from three experimental animal production facilities in Korea were imported to the Daegu-Gyeongbuk Medical Innovation Foundation. Immediately upon arrival, whole blood was collected via jugular vein puncture into the serum-separating tube (SST, BD, 367815, Netherlands). The SST tubes were then centrifuged at 3000 rpm for 10 min at 4°C to separate the serum. The separated serum sample was stored at −70°C until further analysis. The samples were categorized according to age (Table 1).

### Reagents

All enzyme-linked immunosorbent assay (ELISA) and virus detection kits were procured from South Korea. The PRRSV ELISA kit (Bionote PRRS Ab ELISA 4.0; Cat No. EB4404PO) was purchased from BIONOTE (Hwasung, Korea). The PCV2 ELISA kit (VDPro® PCV2 AB ELISA, Cat no. ES-PCV-01) was purchased from MEDIAN, and the PRRSV / PCV2 Detection Kit (Prime-Q, Cat no. ADP-1113Q2) was purchased from GENETBIO (Daejeon, Korea). Reagents from the RNeasy Mini Kit (Qiagen, Hilden, Germany; 74104) and RevertAid First Strand cDNA Synthesis Kit (Thermo Scientific, Basingstoke, UK) were used to prepare the diagnoses.

**Table 1.** The number of animals according to age.

Facility	<1 years	1–2 years	>2 years	Total
A	4	34	7	45
B	6	2	0	8
C	0	4		4

### Serology

All sera were analyzed using ELISA kits: the Bionote PRRS Ab ELISA 4.0, which can detect antibodies against type 1 PRRSV. Samples with sample-to-positive (S/P) ratios of ≥0.4 (cutoff value) were considered positive for the antibody against PRRSV. The VDPro® PCV2 AB ELISA kit can only detect antibodies against PCV type 2. The cutoff value for this ELISA was 0.4, as for the PRRSV ELISA kit.

### Confirmation of infection

Viral RNA was extracted using an RNeasy Mini Kit (Qiagen, USA). The extracted RNA was diluted 2-fold in diethyl pyrocarbonate-treated water. The RNA concentration (ng/μl) and purity were determined using a U-2800 spectrophotometer (Hitachi High Technologies, Japan). Complementary DNA was synthesized from 100 ng of RNA using the RevertAid First Strand cDNA Synthesis Kit (Thermo Scientific, USA) according to the manufacturer’s protocol.

Subsequently, a reaction mixture containing 10 μl of One-step qRT-PCR Premix (Prime-Q PRRSV/PCV2 Detection Kit, GENETBIO), 5 μl (10 pmol) of primer/Probe Mixture of the target gene, and 5 μl of cDNA was subjected to real-time PCR analysis. The primer used to detect the expression of the pathogens and their condition, including annealing temperatures, is listed in Table 2.

### Identification of highly pathogenic PRRSV using PCR

Primers were prepared according to Table 3. Primers encoding the following adhesins: a highly pathogenic form of PRRSV isolated from Korea. Commercial PCR master mixes (AccuPower PCR PreMix, Bioneer, Daejeon, South Korea) were used for amplification; a 20-μl PCR reaction was carried out with the following amplification conditions: 1 cycle at 95°C for 5 min, 35 cycles at 95°C for 20 s, 60°C for 30 s, and 72°C for 30 s (Table 3). The PCR product was visualized on a 1.5% agarose gel at 100 V for 30 min.

**Table 2.** Conditions of real-time PCR analysis.

Step	Temperature (˚C)	Time	Cycle
cDNA synthesis	50	20 min	1
Pre-denaturation	95	10 min	1
Denaturation	95	10 sec	40
Annealing/extension	60	30 sec	

**Table 3.** The primer sequences for PCR.

Target gene	Sequence (5'-3')	Tm (˚C)
JBNU-22-N01	CAGGAACGGTGCTTGTGTTG	60
	TCACCCCAGCTAGTCGATCA	
JBNU-22-N02	ACAGCGAAGGATGCAAGTGA	60
	CACTTGTGAGTGCCAAACCG	
JBNU-22-N03	ACAGCGAAGGATGCAAGTGA	60
	CACTTGTGAGTGCCAAACCG	

## Results

### Serology of PRRSV and PCV antibodies by ELISA

**Figure 1.**
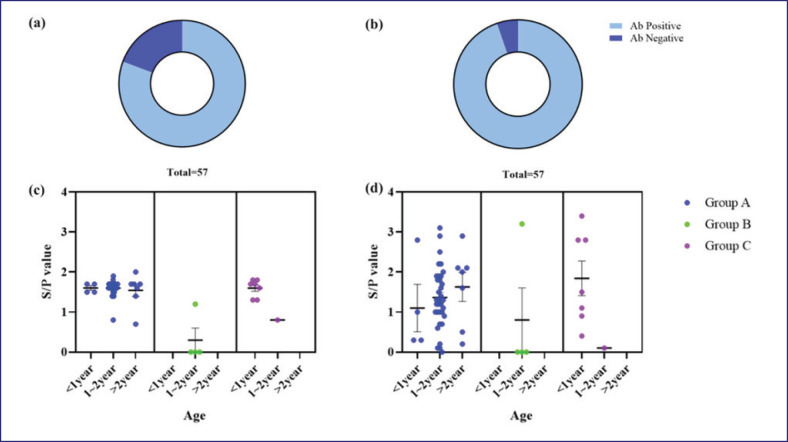
Results of serology. This figure shows PRRSV and PCV antibody positivity rates (a and b) and the distribution of S/P values (c and d).

To determine the formation of PRRSV and PCV antibodies in laboratory swine, all serum samples were analyzed using ELISA (Table 4 and Figure 1). PRRSVs were detected in 46 animals, and 54 pigs showed antibodies against PCVs. The S/P values of ELISA for each group are shown in Figure 1 (c) and (d). Each group was separated by regions of the laboratory animal facility. Of the 57 samples from each group, 46 (80%) were PRRSV antibody-positive tested by ELISA, and 54(95%) were positive for PCVs. Results of PCV are shown in Figure 1 (b) and (d) in terms of contingency tables. As a result of dividing each group by age, no statistically significant differences were identified according to specific age (Figure 1 c and d).

### Confirmation of PRRSV and PCV infection using real-time PCR

Viral infection was confirmed using real-time PCR. In total, 57 serum samples were isolated from laboratory swine in three facilities. According to this test, PRRSV and PCV type 2 were not detected in any animal (Table 5).

### Confirm the infection of NADC34-Like PRRSV by PCR

Outbreaks of NADC34-Like PRRSV have been reported in Korea [21]. Therefore, this study also tried to confirm the NADC34-like PRRSV infection through the PCR, but it was not detected in all of the serums. This ultimately suggests that PRRSV infection was not confirmed in the laboratory animal facility.

## Discussion

This study represents the first investigation of the microbial prevalence in minipigs from experimental animal production facilities. The study focuses on PRRSV and PCV, which cause systemic symptoms, including respiratory issues, in pigs. These viruses are the most prevalent infectious agents on pig farms, causing fever, weight loss, and death upon infection. Particularly in minipigs used in experiments, PRRSV and PCV infection cause significant academic and economic damage [12,13]. Despite the assumption that mini pigs are raised in barrier facilities and are not infected, there have been no studies to prove this in biomedical research.

Official information provided by laboratory animal supply facilities states that minipigs are raised in barrier facilities devoid of vaccinations. According to our findings, the antigen prevalence of PRRSV in minipigs supplied by laboratory animal facilities was 80.7%, whereas that of PCV Type 2 (PCV2) was 94.7% (Table 4). These are higher than the antibody rates (PRRSV: 69.8% and PCV2: 62.3%) of farm pigs in general [15,16]. This discrepancy suggests the possibility of PRRSV or PCV2 infection or vaccination. However, the fact that the antigen positivity rate is 0% may suggest that vaccination is more likely than infection. Additionally, the fact that the subjects were housed in a barrier facility may support this possibility. Nevertheless, the prevalence of antibodies varied among the facilities. For PRRSV, Facility A exhibited a seroprevalence of 84.4%, Facility B exhibited a seroprevalence of 25%, and Facility C exhibited a seroprevalence of 87.5%. Similarly, for PCV2, Facility A had a seroprevalence of 100%, Facility B 25%, and Facility C 100% (Figure 1). However, only one out of four minipigs from Facility B showed antibodies against both PRRSV and PCV2, suggesting lower antibody formation, possibly due to vaccination.

**Table 4.** Number of pigs in which PRRSV and PCV antibodies were detected (*n = *57).

Results	PRRSV	PCV
Positive	46	54
Negative	11	3

Pigs have historically served as valuable animal models for various infectious diseases [22,23]. Vaccines can induce cross-immunity, which is altered by the host immune response, with variations based on sex, genetic factors, and age.

Severe microbial infections in experimental animals can affect parameters such as weight gain and anesthetic depth, potentially causing adverse effects in veterinary management. Pigs are crucial animal models for evaluating vaccine efficacy [22,24,25].

PRRSV and PCV2 have high transmissibility rates and can localize upon infection [9,18]. Given the high infectivity of PRRSV and PCV2 and their persistence following infection, vaccination in experimental animal production facilities may be considered more beneficial than risky. Maintaining pig health through vaccination rather than increasing susceptibility to microbial infections can be advantageous for research involving minipigs. However, when conducting research such as vaccine evaluations, considering cross-immunity plays a pivotal role in preserving the precision and consistency of experimental findings [26,27]. This study revealed that the antibody rates are significantly high in experimental minipigs. Therefore, it is advisable to conduct antibody tests to verify vaccination against prevalent microbes such as PRRSV and PCV2 before starting research.

**Table 5.** Results of PRRSV and PCV detection.

Results	PRRSV	PCV
Positive	0	0
Negative	57	57

Therefore, before conducting research, it is advisable to perform antibody testing to confirm vaccination against microbes prevalent in the country, such as PRRSV and PCV2. For farm pigs, periodic monitoring is necessary to establish epidemiological measures owing to the presence of PRRSV and PCV2. In Korea, surveillance is conducted based on the genotype of PCV2 (PCV2a, PCV2b, PCV2c, and PCV2d) to devise national-level epidemiological measures [16,28].

However, our study did not detect antigens of PRRSV types 1 and 2 or the more pathogenic NADC-like PRRSV and PCV2 (Figure 1). The NADC-like PRRSV of lineage 1 is distinct from the highly pathogenic PRRSV of lineage 8, which is currently observed in China. Because no reports of highly pathogenic PRRSV have emerged in Korea and clinical symptoms were absent in minipigs, additional testing was not pursued. While clinical symptoms may not be evident in cases of singular PRRSV infection, animals with highly pathogenic PRRSV typically exhibit symptoms [29-31]. Thus, the results of this study indicate that PRRSV and PCV infections were not confirmed in the experimental minipigs. This suggests that the breeding and research facility environments are maintained at a reliable level that prevents exposure to infectious agents.

One limitation of this study lies in the restricted sample size, with a relatively high proportion from Facility A (78.9%), followed by Facility C (14.0%) and Facility B (7.0%). However, our study is significant because it is the first to investigate seroprevalence and antigen prevalence in pigs from laboratory animal supply facilities. Future studies should increase the sample size and test for PRRSV and PCV2 antigens in specific organs, such as lymph nodes, during pig necropsy to enhance the reliability of the experimental results.

## Conclusion

This study investigated the seroprevalence and antigen prevalence of PRRSV and PCV in minipigs housed in an experimental animal production facility. Our findings revealed a notable seroprevalence of PRRSV and PCV2 in the minipig population, indicating exposure to these pathogens. Interestingly, the observed antibody prevalence exceeded the levels suggested by the official records of the facility, suggesting potential vaccination practices contrary to the described barrier conditions. Furthermore, significant variations in seroprevalence were observed among facilities, indicating potential differences in management practices or exposure levels. Although vaccination may offer benefits in preventing infections, its implications for research outcomes, particularly in studies involving cross-immunity, require careful consideration. Therefore, we recommend antibody testing for prevalent microbial infections, such as PRRSV and PCV2, before conducting experiments to ensure accurate and reproducible results. This groundbreaking study provides insights into the prevalence of microbial infections in minipigs within laboratory animal production facilities, emphasizing the importance of proactive health management and surveillance to ensure research integrity and animal welfare.

## List of Abbreviations

ELISA, enzyme-linked immunosorbent assay; EU-type, type 1, European prototype; NA-type, type 2, North American prototype; PRRSV, porcine reproductive and respiratory syndrome virus; PCV2, Porcine Circovirus Type 2; PCV1, PCV types 1; PCV2, PCV types 2.
